# *JAK1* as a prognostic marker and its correlation with immune infiltrates in breast cancer

**DOI:** 10.18632/aging.102514

**Published:** 2019-12-02

**Authors:** Bo Chen, Jianguo Lai, Danian Dai, Rong Chen, Xuan Li, Ning Liao

**Affiliations:** 1 Department of Breast Cancer, Cancer Center, Guangdong Provincial People's Hospital, Guangdong Academy of Medical Sciences, Guangzhou, Guangdong 510080, China; 2 Department of Breast Oncology, Sun Yat-Sen University Cancer Center, State Key Laboratory of Oncology in South China, Collaborative Innovation Center for Cancer Medicine, Guangzhou 510060, China

**Keywords:** *JAK1*, breast cancer, prognosis, immune infiltrates, TIL

## Abstract

Clinical trials testing Janus kinase-1 (JAK1) inhibitors in cancers are under way. Whether the *JAK1* mRNA levels in breast tumors correlates with outcome has not been evaluated. *JAK1* expression was analyzed via the Oncomine database and Tumor IMmune Estimation Resource site. Tumor tissues from 57 breast cancer patients were used for qRT-PCR and immune infiltration assessment. *JAK1* expression was significantly lower in breast invasive carcinoma compared with adjacent normal tissues. Public databases (Kaplan-Meier plotter and PrognoScan) showed that low *JAK1* expression was associated with poorer survival. Data from The Cancer Genome Atlas (TCGA) showed that high *JAK1* expression was associated with increased survival in both TNM I-II and TNM III-IV patients. *JAK1* was inversely correlated with tumor size status, lymph node status, and TNM of breast cancer patients. *JAK1* levels were correlated with the T cell transcript-enriched LYM metagene signature and was significantly lower in the low tumor infiltrating lymphocytes (TILs) group. *JAK1* expression levels had significant positive correlations with infiltrating levels of CD8+ T cells, CD4+ T cells, macrophages, neutrophils, and dendritic cells in breast cancer and not with other B cells. In conclusion, *JAK1* mRNA levels were correlated with prognosis and immune infiltrating levels in breast cancer.

## INTRODUCTION

Janus kinases are a family of non-receptor tyrosine kinases which are involved in autoimmune diseases and malignancies [1, 2]. Janus kinase-1 (JAK1) is one of the Janus kinase family members. *JAK1* is essential for IL-6-class inflammatory cytokine signaling, plays a critical role in metastatic cancer progression, and mediates the persistent oncogenic activation of STAT3 in mammary cancer cells that are driven by ERBB2 receptor tyrosine kinase signaling [3]. *JAK1*-deficient cell lines were found to be more tumorigenic than wild-type cells [4]. The evidence indicates that *JAK1* works as either an oncogene or a tumor suppressor under certain conditions or cell contents [5]. Clinical trials testing of JAK1 inhibitors in advanced solid tumors, including breast cancer, are under way [6]. *JAK1* is also expressed in diverse cell types, including immune cells. A recent study has shown that JAK/STAT inhibition acts on the tumor microenvironment to increase production of protumorigenic inflammatory factors in breast cancer patients, which promotes therapeutic resistance [7]. Whether *JAK1* levels in breast cancer tissues are associated with tumor immune infiltrates and clinical outcomes has not been evaluated.

Breast cancer mortality remains the second leading cause of female cancer-related deaths worldwide [8]. Extensive efforts are underway to develop molecular signatures and targeted therapies for specific subsets of breast cancer patients [9]. In recent decades, the prognostic and predictive value of mRNA expression has become more attractive. Studies of the transcriptome, including mRNA levels, in primary breast tumors have been useful for predicting intrinsic subtypes, tumor grade, drug responsiveness, and risk of recurrence, each of which can be used as a prognostic tool [10–12].

Here, we evaluated the association between tumor *JAK1* mRNA levels and breast cancer patients’ prognosis in public databases such as the Kaplan-Meier plotter, PrognoScan database, and the Human Protein Atlas database. Moreover, we also investigated the correlation of *JAK1* mRNA levels with clinicopathological characteristics and tumor-infiltrating immune cells of breast cancer patients. Our findings shed light on the important role of *JAK1* in breast cancer as well as providing a potential relationship and an underlying mechanism between *JAK1* and tumor-immune interactions.

## RESULTS

### The mRNA expression levels of *JAK1*

**Figure 1:**
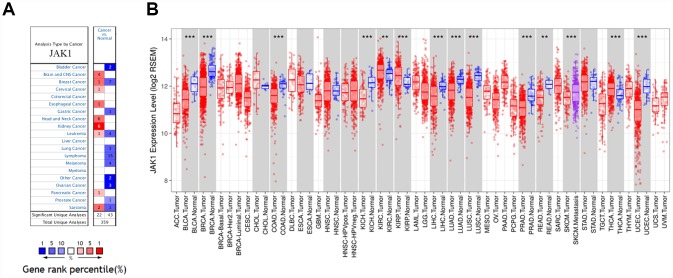
***JAK1* expression levels in human cancers.** (**A**) *JAK1* in data sets of different cancers in the Oncomine database. (**B**) *JAK1* expression levels in different tumor types from TCGA database were determined by TIMER (*P < 0.05, **P <0.01, ***P < 0.001).

The Oncomine database analysis revealed that *JAK1* mRNA expression of breast cancer increased in 1 data set and decreased in 7 data sets compared to the normal tissues (Figure 1A). In addition, *JAK1* mRNA expression was lower in bladder cancer, gastric cancer, lung cancer, ovarian cancer, prostate cancer, melanoma, and lymphoma tumors. Higher expression was observed in brain and CNS, cervical, esophageal, head and neck, kidney, and pancreatic cancers in some data sets. To further evaluate *JAK1* expression of breast cancer, we examined *JAK1* expression using TCGA RNA-sequencing data (Figure 1B). *JAK1* expression was significantly lower in breast invasive carcinoma (BRCA) compared with adjacent normal tissues. The results were similar in basal, HER2+, and luminal breast cancer subtypes.

### *JAK1* mRNA levels predicts prognosis in breast cancer

**Figure 2:**
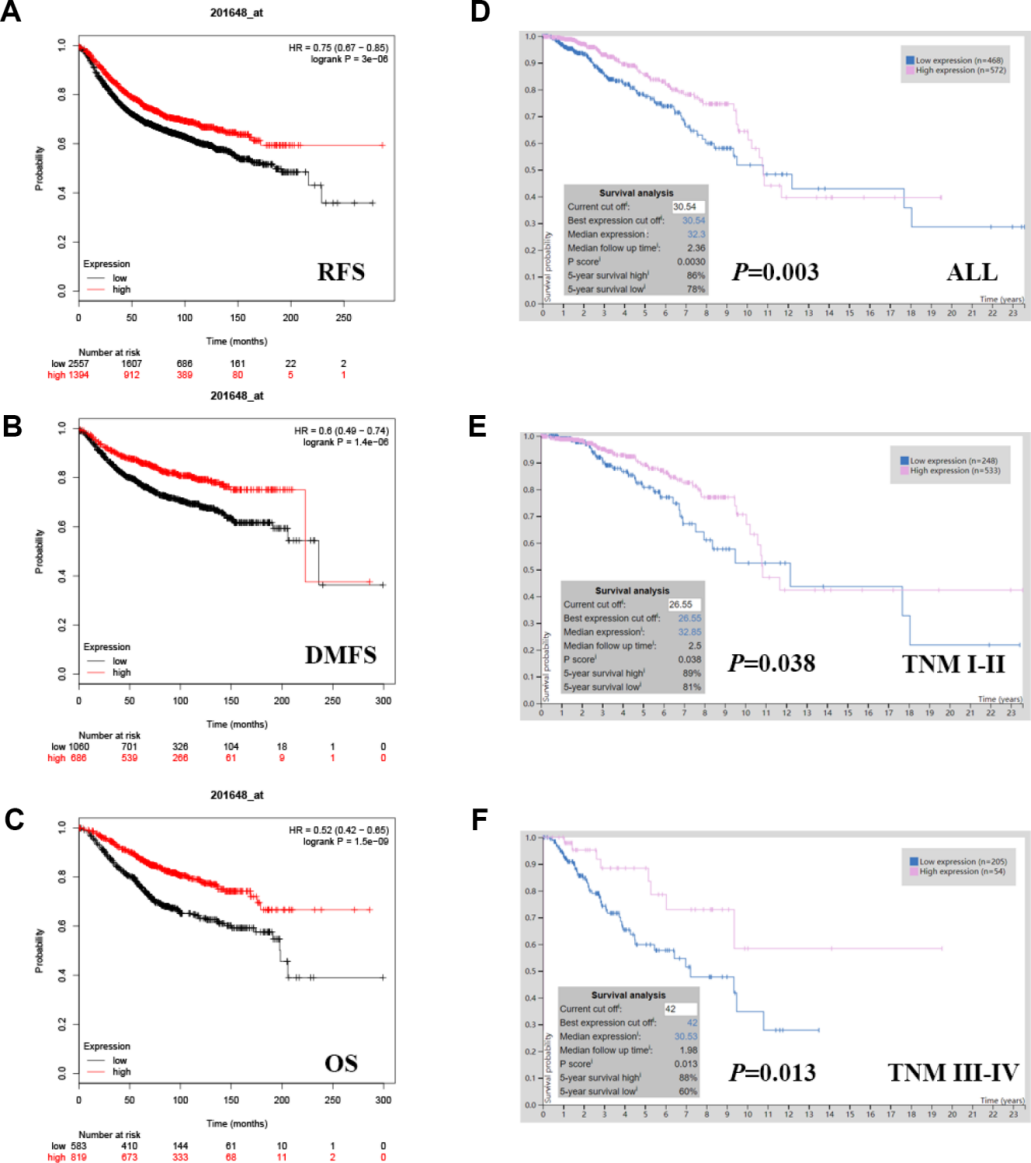
**Kaplan-Meier survival curves comparing the high and low expression of *JAK1* in breast cancer.** In the Kaplan-Meier plotter database, high *JAK1* expression was correlated with good. (**A**) RFS (HR[95% CI] = 0.75[0.67-0.85], P = 3e-06) (**B**) DMFS (HR[95% CI] = 0.6[0.49-0.74], P = 1.4e-06) and (**C**) OS (HR[95% CI] = 0.52[0.42-0.65], P = 1.5e-09). In TCGA data, high *JAK1* expression was also correlated with good OS. (**D**) All breast cancers (P = 0.0030), (**E**) TNM I-II (P = 0.038) (**F**) TNM III-IV (P = 0.013).

*JAK1* expression was evaluated using the PrognoScan (Supplementary Table 1) and was notably found to significantly impact prognosis in breast cancer. Eight cohorts (GSE6532-GPL570, GSE9195, GSE12093, GSE11121, GSE9893, GSE1456-GPL96, GSE3494-GPL96, GSE7390) including different stages of breast cancer showed that high *JAK1* expression was associated with a favorable prognosis (Table 1). Similarly, we also found that *JAK1* expression was associated with a favorable prognosis in breast cancer patients in the Kaplan-Meier plotter database, which is based on Affymetrix microarrays (Figure 2A–2C; RFS HR[95% CI] = 0.75[0.67-0.85], P = 0.0074; DMFS HR[95% CI] = 0.6[0.49-0.74], P = 0.0035; OS HR[95% CI] = 0.52[0.42-0.65], P = 0.0002). In addition, the RNA sequencing data from TCGA were also used to confirm the *JAK1* prognostic value via the Human Protein Atlas database (5-year survival high 86%, 5-year survival low 78%, P = 0.0030, Figure 2D). High *JAK1* expression was associated with increased survival in both TNM I-II (P = 0.038, Figure 2E) and TNM III-IV (P = 0.013, Figure 2F) breast cancer patients. Therefore, it is conceivable that low *JAK1* expression could be a risk factor for a poor prognosis in breast cancer patients.

**Table 1:** Survival analysis of JAK1 mRNA in breast cancer patients (the PrognoScan).

**Dataset**	**Endpoint**	**Number**	**ln(HR-high / HR-low)**	**COX P-value**	**ln(HR)**	**HR [95% CI-low CI-up]**
GSE6532-GPL570	Distant Metastasis Free Survival	87	-1.4754	0.00994975	-1.12962	0.32 [0.14 - 0.76]
	Relapse Free Survival	87	-1.4754	0.00994975	-1.12962	0.32 [0.14 - 0.76]
GSE9195	Distant Metastasis Free Survival	77	-1.47946	0.0298085	-1.45055	0.23 [0.06 - 0.87]
	Relapse Free Survival	77	-1.9106	0.00153696	-1.99732	0.14 [0.04 - 0.47]
GSE12093	Distant Metastasis Free Survival	136	-1.43922	0.00834641	-1.32906	0.26 [0.10 - 0.71]
GSE11121	Distant Metastasis Free Survival	200	-1.44394	7.21E-05	-1.88215	0.15 [0.06 - 0.39]
GSE9893	Overall Survival	155	-1.34982	2.38E-05	-0.85606	0.42 [0.29 - 0.63]
GSE1456-GPL96	Relapse Free Survival	159	-1.57388	0.000196418	-1.8311	0.16 [0.06 - 0.42]
	Disease Specific Survival	159	-1.71397	0.000386357	-2.05196	0.13 [0.04 - 0.40]
	Overall Survival	159	-1.42147	0.00026008	-1.8023	0.16 [0.06 - 0.43]
GSE3494-GPL96	Disease Specific Survival	236	-0.669629	0.0294883	-0.992049	0.37 [0.15 - 0.91]
GSE7390	Distant Metastasis Free Survival	198	-1.99773	0.0381827	-0.565258	0.57 [0.33 - 0.97]
	Relapse Free Survival	198	-0.994522	0.150523	-0.335991	0.71 [0.45 - 1.13]
	Overall Survival	198	-2.58196	0.0381021	-0.585365	0.56 [0.32 - 0.97]

### Correlation of JAK1 expression with the clinicopathological characteristics of breast cancer

The *JAK1* mRNA levels in 57 breast cancer tissues were further correlated with the clinicopathological characteristics of breast cancer. Based on the mean *JAK1* mRNA level, there were 29 patients with high *JAK1* expression and 28 patients with low *JAK1* expression. As shown in Table 2, the expression of *JAK1* was inversely correlated with tumor size status (P = 0.010), lymph node status (P = 0.001), and TNM staging (P = 0.001) of breast cancer patients. No significant correlation was found between *JAK1* expression and other clinicopathological factors, including age (P = 0.357), menopausal status (P = 0.514), histological grade (P = 0.662), ER status (P = 0.516), PR status (P = 0.708), HER2 status (P = 0.248), and breast cancer subtype (P = 0.567).

**Table 2:** Association between JAK1 mRNA and clinicopathological characteristics in breast cancer patients.

**Characteristics**	**JAK1 mRNA**				**P-value**
	**High**		**Low**		
**Age**					0.357
Median (range)	50 (32-74)		51 (31-75)		
≤50 years	17	56.7%	13	43.3%	
>50 years	12	44.4%	15	55.6%	
**Menopausal status**					0.514
Pre	17	54.8%	14	45.2%	
Post	12	46.2%	14	53.8%	
**Tumor size status**					**0.010a**
T1	12	75.0%	4	25.0%	
T2	14	46.7%	16	53.3%	
T3	3	60.0%	2	40.0%	
T4	0	0.0%	6	100.0%	
**Lymph nodes status**					**0.001a**
N0	21	75.0%	7	25.0%	
N1	5	50.0%	5	50.0%	
N2	2	15.4%	11	84.6%	
N3	1	16.7%	5	83.3%	
**TNM staging**					**0.001**
I-II	25	67.6%	12	32.4%	
III	4	20.0%	16	80.0%	
**Histological grade**					0.662
I-II	18	54.5%	15	45.5%	
III	11	45.8%	13	54.2%	
**ER status**					0.516
Negative	10	45.5%	12	54.5%	
Positive	19	54.3%	16	45.7%	
**PR status**					0.708
Negative	9	47.4%	10	52.6%	
Positive	20	52.6%	18	47.4%	
**HER2 status**					0.248
Negative	23	56.1%	18	43.9%	
Positive	6	37.5%	10	62.5%	
**Subtype**					0.567a
HR-/HER2-	6	66.7%	3	33.3%	
HR-/HER2+	3	33.3%	6	66.7%	
HR+/HER2-	16	50.0%	16	50.0%	
HR+/HER2+	4	57.1%	3	42.9%	

a. Using Fisher’s exact test; P < 0.05, statistically significant.

### *JAK1* mRNA levels are associated with tumor infiltrating lymphocytes

**Figure 3:**
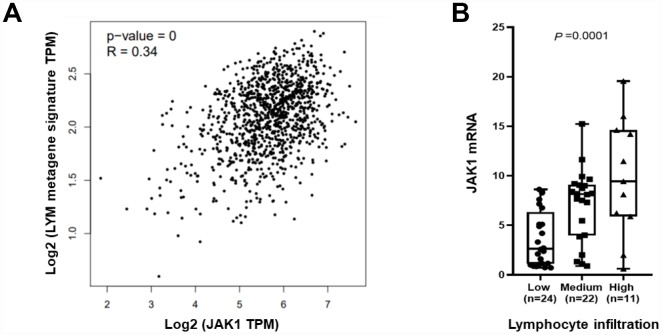
***JAK1* mRNA levels associated with tumor infiltrating lymphocytes**. (**A**) The average expression of the LYM metagene signature (SASH3, CD53, NCKAP1L, LCP2, IL10RA, PTPRC, EVI2B, BIN2, WAS, and HAVCR2) in each breast cancer sample from TCGA is shown relative to *JAK1* mRNA. (**B**) *JAK1* mRNA levels are shown relative to levels of tumor infiltrating lymphocytes in 57 breast cancer samples.

*JAK1* is expressed in immune cells and TILs which have been associated with favorable breast cancer prognosis. A lymphocyte-specific immune recruitment (LYM) metagene signature is related to tumor infiltration by lymphocytes and is associated with favorable prognosis in breast cancer. Therefore, we tested whether breast tumor *JAK1* mRNA levels correlated with the T cell transcript-enriched LYM metagene signature. The results showed that there was a significant correlation between *JAK1* mRNA levels and the LYM metagene in tumor samples from TCGA (Figure 3A). To confirm the correlation, we assessed the presence of TILs in the surrounding stroma of 57 breast cancer cases in which we had already tested the *JAK1* mRNA. The presence of TILs ranged from a score of 0 – 90%. According to the presence of TILs, we divided patients into 3 groups: low TILs (less than 1%, 1% to 9%, and 10% to 19%), medium TILs (20% to 49% and 50% to 74%), and high TILs (75% or greater). There were 24 cases with low TILs (42.1%), 22 cases with medium TILs (38.6%), and 11 cases with high TILs (19.3%). We found that *JAK1* mRNA levels were statistically significantly lower in the low TILs group compared with the high TILs group (Figure 3B, P = 0.0001).

### Correlation analysis between *JAK1* expression and 6 types of infiltrating immune cells

**Figure 4:**
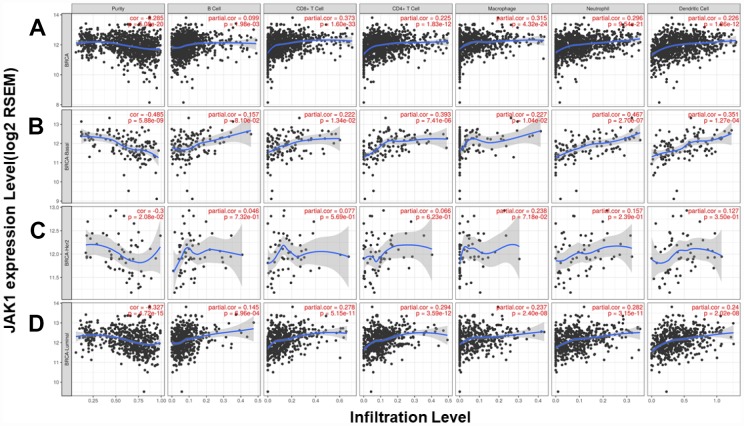
**Correlation of *JAK1* expression with immune infiltration level in the TIMER database**. (**A**) Other than B cells, *JAK1* expression has a significant positive correlation with infiltrating levels of CD8+ T cells, CD4+ T cells, macrophages, neutrophils, and dendritic cells in breast cancer. TIMER database analysis in (**B**) basal, (**C**) HER2 and (**D**) luminal subtypes.

We analyzed the correlation between *JAK1* expression and 6 types of infiltrating immune cells (B cells, CD4 T cells, CD8+ T cells, neutrophils, macrophages, and dendritic cells). The results showed that *JAK1* expression levels had a significantly positive correlation with infiltrating levels of CD8+ T cells (r = 0.373, P = 1.60e-33), CD4+ T cells (r = 0.225, P = 1.83e-12), macrophages (r = 0.315, P = 4.32e-24), neutrophils (r = 0.296, P = 9.54e-21), and dendritic cells (r = 0.226, P = 1.86e-12) in breast cancer and no significant correlations with B cells (r = 0.099, P = 1.98e-03) (Figure 4A). In different breast cancer subtypes, the correlations were not all the same (Figure 4B–4D). In basal-like breast cancer, *JAK1* expression levels were positively correlated with infiltrating levels of CD4+ T cells, neutrophils, and dendritic cells. In luminal breast cancer, *JAK1* expression levels were positively correlated with infiltrating levels of CD8+ T cells, CD4+ T cells, macrophages, neutrophils, and dendritic cells. Since the tumor purity in HER2+ breast cancer was not significant (P = 2.08e-02), the correlations between *JAK1* expression and the 6 types of infiltrating immune cells needs further study to confirm these results.

## DISCUSSION

Here, we report a study supporting the role of *JAK1* in breast cancer. *JAK1* mRNA expression was significantly lower in breast invasive carcinoma compared with adjacent normal tissues. *JAK1* mRNA levels were inversely correlated with the tumor size status, lymph node status, and TNM staging of breast cancer patients. To our knowledge, this is the first study to report a consistent association between increasing *JAK1* mRNA levels and favorable prognosis in breast cancer patients.

*JAK1* is required in the JAK/signal transducer and activator of transcription (STAT) pathway for the activation of STAT1 and STAT2 in response to interferon [13] Previous studies have reported that the JAK/STAT pathway had a central role in driving normal and cancer stem cell growth, and deregulation of the pathway was implicated in the promotion of oncogenic phenotypes, including tumorigenesis, proliferation, anti-apoptosis, invasion, angiogenesis, metastasis, and immune evasion [14–16]. In breast cancer, this pathway was altered by the following mechanisms: [17] (1) down-regulation of phosphotyrosine specific phosphatases; [18] (2) down-regulation of negative regulators of STAT; [19] (3) an increase in the amount of IL-6; [20] and (4) activation of other upstream oncogenic pathways, such as c-Src, ERBB1, or PI3K/ mTOR. [21–23]. It is worth noting that JAK kinases mediate signaling for over 20 cytokines, and genomic changes that alter their activity can have diverse effects. For example, loss-of-function *JAK1* mutations are suggestive of immune evasion in multiple cancers [24] and have been reported in patients who are unresponsive to immunotherapies [25]. *JAK1*-deficient mice exhibited perinatal lethality and phenotypes as diverse as defective lymphopoiesis and lack of IFN response [26]. Therefore, high *JAK1* mRNA being a good prognostic marker in breast cancer patients may be due to the importance of *JAK1* in immune system function.

Another important aspect of this study is that *JAK1* was correlated with diverse immune infiltration levels. Through public database analyses, we observed correlations between *JAK1* mRNA and the LYM metagene signature (moderate correlation) and levels of infiltrating immune cells (weak and moderate correlation). In previous studies, when RNA seq data were used to analyze the relationship between gene mRNA levels and LYM metagene signature or infiltrating immune cells, the correlation coefficients were mostly uncorrelated or a weak and moderate correlation. Strong correlations were rare [27, 28] To verify the results of the database analyses, we further explored the correlation between *JAK1* mRNA levels and TILs using our own breast cancer specimens. We found that *JAK1* mRNA levels were statistically significantly lower in the low TILs group compared with high TILs group in our breast cancer patient cohort. This result proved that, to a certain degree, *JAK1* mRNA levels could reflect lymphocyte infiltration in breast cancer, although we did not identify the cell type of infiltrating lymphocytes. In this era of stratified medicine, the development of immunological biomarkers is of increasing importance [29]. Recent studies support the integration of TILs in a clinicopathologic prognostic model for breast cancer patients and confirm the excellent survival of patients with high TILs after adjuvant chemotherapy [30]. While clinical trials testing JAK inhibitors are under way, determining how specific inhibition of the individual *JAK* family members influences the repertoire and antitumor activities of tumor-infiltrating T cells represents an important area for future investigation.

We must acknowledge that there are potential limitations in our analysis. In our own breast cancer samples, we did not include patients who were diagnosed in the past 10 years, which could have been used to estimate survival. We only did analysis on hematoxylin and eosin–stained slides for TIL assessment and did not determine the type of infiltrating cells by immunohistochemistry. The relationship between *JAK1* mRNA and different immune cell types was based on analysis of sequencing data from public databases. Therefore, subsequent experimental verification is required.

## CONCLUSIONS

In summary, our study provides insights into understanding the potential role of *JAK1* mRNA in tumor immunology and its prognostic value. *JAK1* mRNA levels correlated with prognosis and immune infiltrating levels in breast cancer, indicating that it can be used as a prognostic biomarker. The potential for *JAK1* inhibitors to interfere with immune cells should be evaluated.

## MATERIALS AND METHODS

### Oncomine database analysis

*JAK1* gene expression levels in various types of cancers were identified in the Oncomine database (https://www.oncomine.org/resource/login.html) [31]. The threshold was a P-value of 0.01, a fold change of 1.5, a top 10% gene ranking, and the data had to be from mRNA.

### *JAK1* mRNA levels and survival outcomes in public databases

To investigate the prognostic role of *JAK1* mRNA in breast cancer, the Kaplan-Meier plotter [32] (http://www.kmplot.com; P-value < 0.05, FDR< 0.05), PrognoScan database [33] (http://dna00.bio.kyutech.ac.jp/PrognoScan/; the threshold was adjusted to a Cox P-value < 0.05), and the Human Protein Atlas database [34] (http://www.proteinatlas.org; P-value < 0.05) were used to determine the prognostic significance.

### Patients and tissue specimens

Tumor tissues from 57 breast cancer patients were collected between September 2017 and March 2018 at Sun Yat-Sen University Cancer Centre and used for quantitative reverse transcriptase PCR (qRT-PCR) and immune infiltration assessment. Our experiments were in accordance with the ethical standards formulated in the Helsinki Declaration. The Ethics Committee of Sun Yat-Sen University Cancer Centre Health Authority approved this study.

### Quantitative reverse transcriptase PCR

The resected tissues were immediately cut and stored in RNAlater (Ambion, Austin, Texas, USA). Total RNA was extracted using TRIzol reagent (Life Technologies, Carlsbad, CA, USA). cDNA was synthesized from 2.0 *μ*g of total RNA using random hexamers and SuperScript III Reverse Transcriptase (Invitrogen, Carlsbad, CA, USA) following the manufacturer’s protocol. qRT-PCR was performed using SYBR Premix Ex Taq II (Takara Bio Inc, Otsu, Japan). The housekeeping gene HMBS was used as an endogenous control. Primer information: JAK1: 5′-CCACTACCGG ATGAGGTTCTA-3' (forward) and 5'-GGGTCTCGA ATAGGAGCCAG-3' (reverse); HMBS: 5′-GGCAATG CGGCTGCAA-3' (forward) and 5'- GGGTACCCAC GCGAATCAC-3' (reverse). Relative quantification was calculated as 2^-ΔCt^. ΔC_t_ values = target gene mean C_t_ value - control gene mean C_t_ value [27].

### Assessment of immune infiltration

Stromal lymphocytic infiltration was defined as the percentage of tumor stroma containing infiltrating lymphocytes, hereinafter referred to as “TIL,” which was similar to a previous publication [35]. Intra-tumoral TILs were not included in this study. According to the guidelines for TIL assessment in breast carcinoma [36, 37], analysis on hematoxylin and eosin–stained slides were independently evaluated by 2 board certified breast pathologists. We used semi continuous variables (deciles): less than 1%, 1% to 9%, 10% to 19%, 20% to 49%, 50% to 74%, and 75% or greater [38]. All cases for which the discordance between the 2 pathologists reached greater than 15% were reviewed together until a consensus was reached.

### TIMER database analysis

We analyzed *JAK1* expression and the correlation of *JAK1* expression with the abundance of 6 types of infiltrating immune cells (B cells, CD4+ T cells, CD8+ T cells, neutrophils, macrophages, and dendritic cells) in breast cancer patients via The Tumor IMmune Estimation Resource (TIMER) algorithm database (https://cistrome.shinyapps.io/timer/). [39] Tumor purity is a vital factor that influences the analysis of immune infiltration in tumor samples by genomic approaches. [40]

### Gene correlation analysis in GEPIA

The online database Gene Expression Profiling Interactive Analysis (GEPIA) (http://gepia.cancer-pku.cn/index.html) was used to find the significant correlation of *JAK1* expression with a lymphocyte-specific immune recruitment (LYM) metagene signature. Gene expression correlation analysis was performed on The Cancer Genome Atlas (TCGA) expression data. The LYM metagene was part of the winning prognostic model in the Sage Bionetworks DREAM breast cancer prognosis challenge [41, 42].

### Statistical analysis

The Chi-squared test and Fisher’s exact test were used to investigate the significance of the correlation of *JAK1* expression with clinicopathological features in breast cancer via SPSS for Windows version 23.0 (Chicago, IL, USA). ANOVA was used to identify the *JAK1* mRNA levels in different TIL groups. The correlation of gene expression was evaluated using the Spearman's correlation coefficient. The strength of the correlation was determined using the following guide for the absolute value: 0.00–0.29 (weak), 0.30–0.59 (moderate), 0.60–0.79 (strong), and 0.80–1.0 (very strong). P-values < 0.05 were considered statistically significant. [28]

## Supplementary Material

Supplementary Table 1
